# Predicting Outcome for Early Attention Training After Acquired Brain Injury

**DOI:** 10.3389/fnhum.2022.767276

**Published:** 2022-05-18

**Authors:** Aniko Bartfai, Mattias Elg, Marie-Louise Schult, Gabriela Markovic

**Affiliations:** ^1^Department of Clinical Sciences, Danderyd University Hospital, Karolinska Institutet, Stockholm, Sweden; ^2^Division of Rehabilitation Medicine, Danderyd University Hospital, Stockholm, Sweden; ^3^Department of Management and Engineering, IEI, Linköping University, Linköping, Sweden

**Keywords:** attention training, prediction, early rehabilitation, functional outcome, acquired brain injury, statistical process control (SPC)

## Abstract

**Background:**

The training of impaired attention after acquired brain injury is central for successful reintegration in daily living, social, and working life. Using statistical process control, we found different improvement trajectories following attention training in a group of relatively homogeneous patients early after acquired brain injury (ABI).

**Objective:**

To examine the contribution of pre-injury factors and clinical characteristics to differences in outcome after early attention training.

**Materials and Methods:**

Data collected in a clinical trial comparing systematic attention training (APT) with activity-based attention training (ABAT) early after brain injury were reanalyzed.

**Results:**

Stroke patients (*p* = 0.004) with unifocal (*p* = 0.002) and right hemisphere lesions (*p* = 0.045), and those with higher mental flexibility (TMT 4) (*p* = 0.048) benefitted most from APT training. Cognitive reserve (*p* = 0.030) was associated with CHANGE and APT as the sole pre-injury factor. For TBI patients, there was no statistical difference between the two treatments.

**Conclusion:**

Our study identifies indiscernible factors predicting improvement after early attention training. APT is beneficial for patients with right-hemispheric stroke in an early recovery phase. Knowledge of prognostic factors, including the level of attention deficit, diagnosis, and injury characteristics, is vital to maximizing the efficiency of resource allocation and the effectiveness of rehabilitative interventions to enhance outcomes following stroke and TBI.

## Introduction

Cognitive impairment is one of the major limiting factors for a successful recovery after acquired brain injury (ABI), leading to constraints in resuming earlier social roles in the home, personal relations, and working life. This issue is particularly pronounced in persons with primarily cognitive injuries with minor or no motor impairment (Skoglund et al., 2019). In addition, the family, social, and working environment may set expectations that cannot be met by the brain-injured person leading to misunderstandings, personal failures, and marginalization (Wallenbert and Jonsson, 2005; Arntzen et al., 2015; Nasr et al., 2016; Goverover et al., 2017).

Attention dysfunction remains one of the most common long-term cognitive consequences after brain injury affecting learning skills, daily functioning, and the social and emotional life of the individual (Barker-Collo et al., 2010; Ponsford et al., 2014; Markovic et al., 2020).

There are several ways to remedy attention impairment, including structured restorative attention training tapping on underlying processes (Raskin, 2011; Cicerone et al., 2019), compensatory adaptation to activity requirements allowing a gradually resumed independence (Raskin, 2011) and meta-cognitive strategy training to improve generalization of learned skills (Sohlberg and Turkstra, 2011; Dymowski et al., 2016; Cicerone et al., 2019).

Several guidelines recommend focusing attention on a high priority level (Haskins et al., 2013; Ponsford et al., 2014; Cicerone et al., 2019). The method Attention Process Training (APT) (Sohlberg and Mateer, 1989) is considered a practice standard, i.e., should regularly be used to ameliorate attention deficits in the chronic stage after brain injury (Haskins et al., 2013; Cicerone et al., 2019).

Studies regarding effects during the acute stage show more varying results due to difficulties separating the effects of spontaneous recovery and attention training (Cicerone et al., 2005; Barman et al., 2016).

We have used Statistical Process Control (SPC) with repeated assessments as an alternative approach to pre-and post-evaluation to study the effects of attention training during the first 4 months post-ABI (Markovic et al., 2017a; Markovic et al., 2020). SPC is a set of statistical methods that can follow a rehabilitation process over time to detect either deteriorating performance or improvements (Montgomery, 2012). SPC combines statistical rules with time-series graphical representations enabling decision-making for professionals in clinical practice (Callahan and Barisa, 2005).

This study examines possible predictive factors advantageous for individual treatment outcomes. Although APT is recommended as a practice standard, little is known of the predictive factors.

We performed a PubMed literature search (predictor, prognosis, cognitive rehabilitation, cognitive training, and brain injury), yielding only 16 studies, of which two were relevant (Rosti-Otajärvi et al., 2013; Tornås et al., 2019). There are no studies on predictive factors on attention training. Different features for the participants, such as age, sex or demographical variables, occupational status and premorbid cognitive level, might be considered pre-injury characteristics. Another group of mechanisms possibly influencing a treatment might be clinical variables, such as aetiology and localization of a lesion. Further, the outcome of a rehabilitative treatment might depend on the initial cognitive state, measured as performance on tests of attention, mental flexibility, and speed.

The present explorative study aimed to identify the impact of those factors, such as injury-related clinical factors, initial cognitive functioning, and pre-injury variables for improvement after the two different types of intensive attention training.

## Materials and Methods

This work is part of a clinical RCT trial (registration no. NCT02091453) (Bartfai et al., 2014) comparing two attention interventions approved by the Karolinska Institutet Ethical Committee (registration no 2007/1363-31).

### Participants

In- or outpatients (*n* = 59) (Table 1) with mild to moderate stroke or traumatic brain injury participated from a university hospital providing certified brain injury rehabilitation programs according to the Commission on Accreditation of Rehabilitation Facilities (CARF). All patients had a Barthel index >50 points (Mahoney and Barthel, 1965), Matrices (WAIS-III) ≥ 7 scaled score and Albert’s Test ≤ 3 errors (Lezak et al., 2012), Rivermead Behavioural Memory Test, profile score ≥ 10, screening score ≥ 2 (Wilson et al., 2000), and score <10 on the Hospital Anxiety and Depression Scale (Zigmond and Snaith, 1983). Only 8% had suffered a previous brain injury.

**TABLE 1:** Demographical characteristics of participants at baseline assessment according to the type of treatment and treatment outcome (CHANGE/NO CHANGE).

Variable	Total sample	APT		ABAT	
		CHANGE	NO CHANGE	CHANGE	NO CHANGE
		
	(*n* = 59)	(*n* = 27)	(*n* = 5)	(*n* = 15)	(*n* = 12)
Age, years, mean ± SD	45 ± 11	45 ± 13	42 ± 9	44 ± 12	46 ± 8
Gender Female, *n* (%)	21 (36)	13 (48)	2 (40)	4 (27)	3 (25)
**Marital status, *n* (%)**					
Married/co-habiting	50 (85)	21 (78)	5 (100)	13 (87)	9 (75)
Single	9 (15)	6 (22)	0 (0)	2 (13)	3 (25)
**Education*^a^*, *n* (%)**					
<9 years	1 (2)	1 (4)	0 (0)	0 (0)	0 (0)
10–12 years	15 (25)	7 (26)	1 (20)	4 (27)	4 (33)
13–15 years	29 (49)	12 (44)	3 (60)	6 (40)	7 (58)
**Occupational skills level*^b^*, *n* (%)**					
Level 1	4 (7)	1 (4)	0 (0)	2 (13)	1 (8)
Level 2	13 (22)	8 (30)	0 (0)	3 (20)	2 (17)
Level 3	20 (34)	6 (22)	4 (80)	4 (27)	6 (50)
Level 4	22 (37)	12 (44)	1 (20)	6 (40)	3 (25)
HADS, Depression, M(q1–q3)	3(1–6)	3(1–6)	6(2–7)	5(1–9)	3(0–7)
HADS, Anxiety, M(q1–q3)	5(1–7)	5(1–7)	5(2–9)	5(2–8)	3(1–6)
Matrices (WAIS-III)*^c^*	17 ± 4	17 ± 4	18 ± 3	17 ± 3	16 ± 4
**Cognitive reserve (CR)*^d^*, *n* (%)**					
Low CR	17 (29)	8 (30)	3 (60)	2 (13)	4 (33)
High CR	42 (71)	19 (74)	2 (40)	13 (87	8 (67)

*APT, Attention Process Training; ABAT, Activity-based attention training; n, number; HADS, Hospital Anxiety and Depression Scale; (WAIS-III), Wechsler Adult Intelligence Scale-III.*

*^a^Completed years of highest attained educational level, from elementary school to master’s degree and above.*

*^b^Occupational skill level as defined by the International Standard Classification of Occupation, ISCO-08 and categorized in order of increasing complexity in task requirements.*

*^c^Number of correct items on Matrices (WAIS-III), raw scores.*

*^d^Low CR is defined as low scores on at least 2 of CR measures, accordingly high CR is defined as high scores on at least 2 of CR measures.*

Attention impairment was defined as a performance ≤70% on ≥2 subtests of the APT test with a margin for expected daily fluctuations, lower than the recommended cut-off at 80% (Sohlberg and Mateer, 1987). Mean values and frequencies for demographic characteristics are presented in Table 1.

### Treatments

Intensive interdisciplinary rehabilitation (6 h/day, 4–5 days weekly, 8–12 weeks) was complemented by 20 h of additional attention training (3–5 days weekly, 4–6 weeks) within 4 months post-injury (*M* = 60, *SD* = 26 days; min = 16; max = 105). Participants were randomized to APT (45–90 min/session), or Activity-Based Attention Training (ABAT) (60–120 min/session).

The APT (Sohlberg and Mateer, 1987) is a process-oriented hierarchically based, person-centred, individual attention-training program comprising repetitive exercises, meta-cognitive strategy training, and psycho-education.

The ABAT (Stephens et al., 2015; Sargénius Landahl, 2021) involves attention-demanding activities in the domains of personal care, household activities, work, and leisure. By integrating performance skills training and metacognitive training, attempts are made to incorporate the International Classification of Functioning and Health (ICF) domains body functions with activity and participation (Sigmundsdottir et al., 2016).

### Measurements

#### The Dependent Variable

The dependent variable in the present study was CHANGE/NO CHANGE. CHANGE comprises patients with significant improvement in performance for Paced Auditory Serial Addition Test (PASAT) (Gronwall, 1977) according to statistical rules for SPC. The basis for identifying such improvements originates in repeated-hypothesis testing, where each observation in the time series data is evaluated against a probability distribution (Callahan and Barisa, 2005; Montgomery, 2012). Rules for identification of significant improvements were: (i) one data-point outside 3 SD from the mean, and (ii) 2 of 3 consecutive points falling ≥2 SD from the centreline (Benneyan et al., 2003). NO CHANGE patients displayed fluctuations between treatment sessions but no significant improvement. Parts of these data (PASAT) were already discussed previously (Markovic et al., 2017b; Markovic et al., 2019). The present study differs by using the dichotomized variables CHANGE/NO CHANGE as the dependent variable.

Paced Auditory Serial Addition Test (PASAT) was selected as the primary outcome variable since the SPC design required a minimum of eight repeated measurements. The test was administered before the attention training, after every 3rd h of treatment and after completing therapy (Bartfai et al., 2014). This test fulfilled the requirements for an acceptable range of difficulty and repeatability. In addition, learning effects (Gronwall, 1977) were analyzed. Although performance improvement was found in both groups during the first three administrations, statistical differences in performance emerged from the fourth administration (Markovic et al., 2019).

#### The Independent Variables

The independent variables were pre-injury, injury-related, and initial cognitive functioning (baseline assessment).

##### Pre-injury Variables

Age, gender, living conditions, length of education, and occupational skills were selected as pre-injury variables.

Furthermore, premorbid cognitive functioning, often estimated as premorbid IQ, is also a crucial pre-injury variable (Lezak et al., 2012). Lately, the measure of Cognitive reserve (CR) has been favored since it reflects factors influencing cognitive processes during the lifetime (Barulli and Stern, 2013; Oldenburg et al., 2016; Umarova et al., 2019). CR is usually estimated by a combination of premorbid cognitive function occupational and educational level in contrast to parameters related to brain reserve, such as intracranial volume. There are no uniform ways to estimate CR since countries’ occupational requirements and educational systems vary (Umarova et al., 2019). Procedure and statistics underlying CR are presented in Supplementary Appendix 1.

##### Injury-Related Variables and Initial Cognitive Functioning

Injury-related variables were classified into (1) distribution according to the number of lesions (unifocal, multifocal corresponding to ≥2 lesions), (2) localization according to the side of injury (right, left, and bilateral), and (3) distribution according to the lesion site (anterior, posterior, subcortical, and global). The classification was based on medical and imagining records and performed jointly by a rehabilitation specialist and a neuropsychologist.

The APT test (Sohlberg and Mateer, 1987) screens attention dysfunction. The task is to sustain focus and respond to auditory and visual stimuli presented in distractor conditions for focused, sustained, selective, divided, and alternating attention. Performance scores for each subtest are presented as the percentage of correct responses within the timeframe. In addition, a weighted mean score was formed comprising the results from all subtests <70%.

Digit Span (forward) and Letter-number sequencing from the Weschler Adult Intelligence Scale (WAIS-III) (Lezak et al., 2012) and Spatial Span (forward) from the Wechsler Memory Scale III (WMS III) (Lezak et al., 2012) required reproduction of sequences of stimuli. Therefore, they were considered as measures of verbal and visual working memory. Data are presented in raw scores. High scores indicate better performance.

The Colour-Word Interference Test (CWIT) from Delis-Kaplan Executive Function Test (D-KEFS) (Delis et al., 2001) is a four-part test measuring processing speed, the ability to inhibit cognitive interference, and verbally mediated cognitive flexibility. It requires naming colours presented in different settings. Low scores indicate better performance.

The Trail Making Test (TMT) from D-KEFS (Delis et al., 2001) is a five-part test measuring visual scanning, processing speed, and visually mediated cognitive flexibility by sequencing stimuli as fast as possible. In this study, we present data for subtests 2–5. Again, lower scores indicate better performance.

Ruff 2&7 Selective Attention Test (Ruff 2&7) (Ruff et al., 1992) measures sustained and selective visual attention. The subject is required to detect the numbers 2 and 7 embedded in sections of letters and digits. Data are presented in *T* scores and corrected for age and education level. Higher scores indicate better performance.

The Rey Auditory Verbal Learning Test (RAVLT) (Lezak et al., 2012) is a verbal learning test distinguishing between different memory components. The subject is asked to repeat a list of 15 words presented five times and recall the list with a 30-min delay. Raw scores were transformed to *T* scores using age- and gender corrected normative data (Lezak et al., 2012). High scores indicate better verbal learning and memory.

### Statistical Methods

Statistical process control combines statistical rules with time-series graphical representations in control charts allowing for change detection in an ongoing intervention before results from a more extensive, ex-post evaluation are available (Callahan and Barisa, 2005; Thor et al., 2007). With I-diagrams (individual diagrams), the RCT study design in (Markovic et al., 2017a) allowed the identification of participants with significant improvement (CHANGE) from those who did not improve (NO CHANGE). The following procedure was used:

(1) I-diagrams within SPC was used to construct a dichotomous variable as CHANGE/NO CHANGE. Treatment outcome was based on individual control charts where CHANGE was defined according to rules described previously (Supplementary Figure 1).
(2) Exploratory data analysis (EDA) was performed, examining differences between treatments for pre-injury variables, injury-related variables, and initial cognitive functioning post-ABI.
(3) Multiple linear regression was used to analyze the relationship between identified significant correlations and CHANGE/NO CHANGE (constant).
(4) Differences between treatments were formally evaluated with ANOVA (parametric tests) and chi-square (non-parametric tests). In addition, effect sizes were analyzed to assess the magnitude of differences.

I-diagrams are based on the mean (Centreline) and three standard deviations as the upper and lower control limit, respectively. The control chart was adapted to fit the clinical needs of early neurorehabilitation baseline measurement. Therefore, the two first observations were used to define the centre line. Next, a pooled SD measure based on the two first observations was used to calculate control limits (Markovic et al., 2017a).

As an EDA, we have first explored the relationship between each test and CHANGE/NO CHANGE using Pearson Correlation Coefficient for neuropsychological tests. Then, to further investigate the linkage between CHANGE/NO CHANGE and neuropsychological tests, significant correlations were explored first in multiple linear regression analysis and then in ANOVAs with separated interaction variables, i.e., in two separate data sets, ABAT and APT.

Based on the requirements for ANOVA, CHANGE/NO CHANGE was treated as the independent variable and neuropsychological test scores as dependent variables.

Eta square analysis for ANOVA, η^2^ (small ∼ 0.01, medium ∼ 0.06, and large ≥0.14) was made to assess the effect size (Lakens, 2013). Chi-square tests were used for analysis on pre-injury and injury-related variables. Effect sizes (ES) (Cohen, 1988) were calculated according to Cramer’s V (ES V) (weak ≤0.2, moderate = 0.2–0.6, and strong ≥0.6). We have used odds-ratios (OR) for the Chi-Square analysis to estimate the odds for obtaining CHANGE, given the presence of independent variables.

The statistical software used for all statistical analysis was SPSS for Windows version 22.0 and MS Excel. Statistical significance level was set at *p* < 0.05 2-tailed for all analyses.

## Results

**FIGURE 1:**
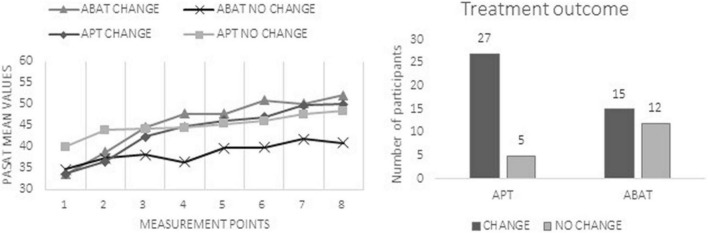
The left chart presents mean scores of the Paced Auditory Serial Addition Test (PASAT) at each measurement point. These data were published earlier (Markovic et al., 2020) and are enclosed to illustrate the different improvement patterns in performance during the study. The right chart shows the distribution of patients in the CHANGE vs. NO CHANGE groups separated according to treatment groups (APT, Attention Process Training; ABAT, Activity-Based Attention Training).

The odds for obtaining CHANGE were 4.3 times higher for APT patients than for ABAT patients [OR of 0.231 for NO CHANGE/CHANGE with 95% CI (0.068, 0.784)] (Figure 1). The effect size was moderate (ES V = 0.317). The *X*^2^ statistics [Pearson *X*^2^ (1, *N* = 59) = 5,930, *p* = 0.015; Fisher’s Exact test = 0.021] were published earlier (Markovic et al., 2020). In the following, we present the contribution of injury-related, cognitive, and demographical variables to the observed differences.

### Injury-Related Clinical and Cognitive Variables

Frequencies for aetiology and injury-related characteristics are presented in Table 2.

**TABLE 2:** Injury related characteristics of participants at baseline assessment according to type of treatment and treatment outcome (CHANGE vs. NO CHANGE).

Variable	Total sample	APT		ABAT	
		CHANGE	NO CHANGE	CHANGE	NO CHANGE
		
	(*n* = *59*)	(*n* = *27*)	(*n* = *5*)	(*n* = *15*)	(*n* = *12*)
Etiology stroke*, *n* (%)	46 (78)	22 (82)	4 (80)	9 (60)	11 (92)
**Injury distribution, *n* (%)**					
Focal	29 (42)	15 (56)	0 (0)	7 (47)	7 (58)
Multifocal (≥2)	34 (58)	12 (44)	5 (100)	8 (53)	5 (42)
**Injury side, *n* (%)**					
Left hemisphere	25 (42)	7 (26)	4 (80)	7 (47)	7 (58)
Right hemisphere	22 (37)	14 (52)	1 (20)	5 (33)	3 (25)
Bilateral	12 (20)	6 (22)	0 (0)	3 (20)	2 (17)
**Lesion site, *n* (%)**					
Anterior	20 (34)	6 (22)	1 (20)	7 (47)	6 (50)
Posterior	11 (19)	7 (26)	1 (20)	1 (7)	2 (17)
Subcortical	22 (37)	9 (33)	3 (60)	6 (40)	4 (33)
Global	6 (10)	5 (19)	0 (0)	1 (7)	0 (0)

*For stroke patients, thrombosis accounted for 48%. TBI was a result of traffic accidents (n = 6), winter sports (n = 3), falls from heights (n = 3), and assault of person (n = 1).

**FIGURE 2:**
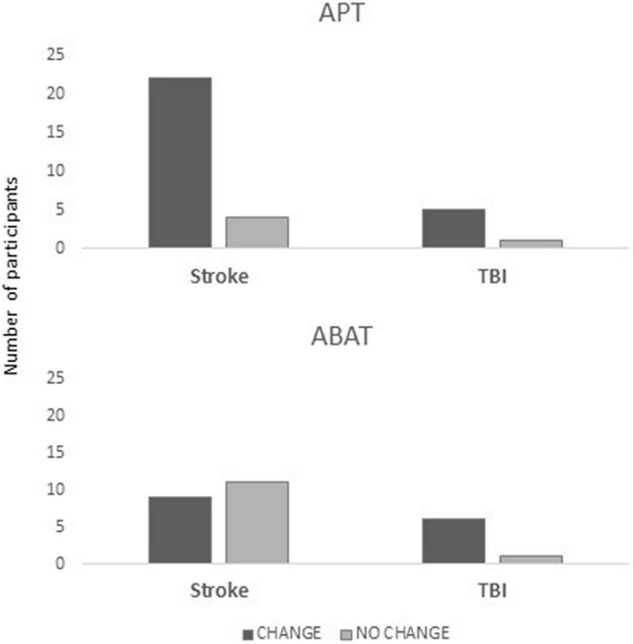
Distribution of patients in the CHANGE vs. NO CHANGE groups split for diagnosis (stroke, TBI) and treatment (APT, Attention Process Training; ABAT, Activity-Based Attention Training), where the y-axis indicates the number of patients in each subgroup.

Analysis between diagnostic groups showed that significantly more stroke patients obtained CHANGE with APT training [Pearson *X*^2^ (1, *N* = 46) = 8,073, *p* = 0.004; Fisher’s Exact test = 0.010; ES V = 0.419; OR of 0.149 for NO CHANGE/CHANGE with 95% CI (0.039, 0.593)] (Figure 2). Among TBI patients, there was no difference in the distribution of patients obtaining CHANGE depending on the type of intervention.

**FIGURE 3:**
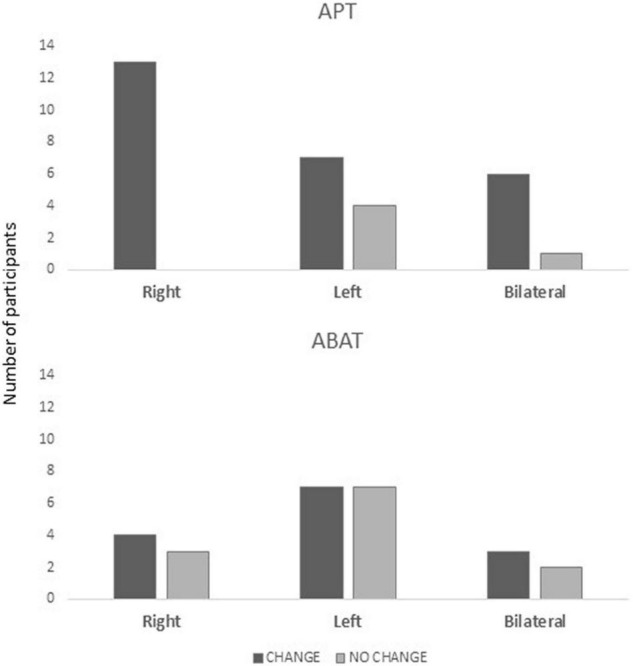
Distribution of patients in the CHANGE vs. NO CHANGE groups split for injury localization (right, left, and bilateral) and treatment (APT, Attention Process Training; ABAT, Activity-Based Attention Training), where the y-axis indicates the number of patients for each subgroup.

Comparison between unifocal and multifocal lesions showed that APT training was more beneficial for patients with unifocal lesions [Pearson *X*^2^ (1, *N* = 59) = 9,886, *p* = 0.002; Fisher’s Exact test = 0.002; ES V = 0.584; OR = 0.318 for NO CHANGE/CHANGE with 95% CI (0.173, 0.587) *p* = 0.002]. All patients with right hemisphere lesions benefitted from APT treatment [Pearson *X*^2^ (1, *N* = 22) = 6,079, *p* = 0.014; Fisher’s Exact test = 0.036; ES V = 0.526] (Figure 3). There were no significant differences for lesion sites (anterior, posterior, subcortical, and global). None of the injury-related variables was related to CHANGE in the ABAT group.

Other post ABI variables were initial neuropsychological test results (Table 3). EDA with Pearson Correlation analysis revealed a significant correlation between CHANGE/NO CHANGE verbal mental flexibility (TMT 4), *p* = 0.016 and visual mental flexibility (CWIT 4), *p* = 0.026. Multiple linear regression was used to test if these neuropsychological variables also significantly predicted CHANGE. The fitted regression model was: CHANGE/NO CHANGE = 0.911 + 0.002*(visual mental flexibility, TMT 4) – 0.002*(verbal mental flexibility, CWIT 4). The overall regression was statistically significant [*R*^2^ = 0.120, *F*_(2,55)_ = 3.761, *p* = 0.029]. However, we found that neither verbal mental flexibility (β = 0.002, *p* = 0.143) nor visual mental flexibility alone (β = 0.002, *p* = 0.376) significantly predicted CHANGE.

**TABLE 3:** Neuropsychological characteristics of participants at baseline assessment according to the type of treatment and treatment outcome (CHANGE/NO CHANGE).

Variable		APT		ABAT	
	Total sample	CHANGE	NO CHANGE	CHANGE	NO CHANGE
	(*n* = *59*)	(*n* = *27*)	(*n* = *5*)	(*n* = *15*)	(*n* = *12*)
**APT test*^a^*, % of correct responses**					
Weighted APT test score	40 (15)	36 (13)	49 (10)	45 (17)	41 (17)
Focused attention	94 (13)	93 (18)	97 (4)	93 (8)	96 (6)
Sustained attention	45 (20)	39 (18)	57 (20)	55 (24)	39 (16)
Selective attention	45 (22)	39 (22)	50 (13)	50 (17)	50 (27)
Divided attention	89 (13)	89 (13)	93 (12)	89 (11)	89 (19)
Alternating attention	36 (24)	32 (22)	46 (14)	38 (26)	39 (29)
**Working Memory Index Scale (WAIS-III)*^b^*, number of correct answers**					
Digit Span (forward)	9 (2)	9 (2)	9 (3)	8 (2)	9 (2)
Spatial Span	8 (2)	8 (2)	8 (2)	8 (2)	7 (2)
Letter-Number Sequencing	9 (3)	10 (3)	10 (2)	9 (2)	9 (3)
**Trail Making Test (TMT)*^d^*, seconds**					
Number sequencing (TMT 2)	42 (25)	43 (24)	40 (30)	39 (13)	47 (36)
Letter sequencing (TMT 3)	46 (31)	44 (29)	76 (76)	38 (15)	49 (15)
Number-letter switching (TMT 4)	104 (56)	97 (49)	146 (126)	87 (25)	126 (51)
Motor speed (TMT 5)	30 (20)	24 (8)	34 (14)	32 (26)	41 (32)
**Color-Word Interference Test (CWIT)*^c^*, seconds**					
Colour naming (CWIT 1)	36 (10)	35 (8)	34 (11)	37 (7)	40 (17)
Word reading (CWIT 2)	27 (6)	26 (6)	25 (5)	26 (6)	29 (8)
Inhibition (CWIT 3)	64 (25)	58 (13)	56 (12)	69 (29)	77 (37)
Inhibition/switching (CWIT 4)	78 (29)	70 (16)	92 (43)	79 (25)	91 (42)
**Ruff 2&7 Selective Attention Test, *T* score**					
Automatic Detection Speed	44 (10)	46 (12)	42 (15)	45 (8)	42 (8)
Controlled Search Speed	41 (10)	42 (12)	40 (12)	43 (9)	39 (8)
Total Speed	44 (10)	46 (12)	43 (14)	45 (8)	41 (8)
Automatic Detection Accuracy	45 (12)	44 (12)	47 (15)	48 (9)	43 (13)
Controlled Search Accuracy	41 (12)	41 (12)	45 (15)	42 (13)	39 (12)
Total Accuracy	43 (11)	42 (11)	47 (15)	45 (10)	41 (12)
**Rey Auditory Verbal Learning Test*^d^*, *T* score**					
Immediate recall	43 (13)	43 (12)	43 (20)	40 (13)	44 (14)
Delayed recall	41 (16)	41 (15)	49 (21)	40 (15)	40 (19)

*The results are presented with mean values and standard deviations based on raw scores.*

*^a^The attention process training test (APT) measures attention dysfunction regarding focused, sustained, selective, divided, and alternating attention. The weighted APT test score comprises individual mean values for subtests with results of <70% correct responses.*

*^b^The wechsler adult intelligence scale-IV (WAIS-IV).*

*^c^The trail making test and the colour-word interference test are parts of the Delis-Kaplan executive function system (D-KEFS).*

*^d^Immediate recall comprises the total number of correct responses after five trials in T scores. The delayed recall includes the number of recalled words after a 30-min time-lapse in T scores.*

Differences between treatments were analysed with one-way ANOVA. For APT patients, ANOVA indicated that CHANGE was associated with better performance on inhibition/switching (CWIT 4) *F*_(1,31)_ = 4,245, *p* = 0.048, η^2^ = 0.128, 95% CI [0,000, 0.349].

For ABAT patients, ANOVA showed that CHANGE was associated with low scores on mental flexibility (TMT 4) *F*_(1,26)_ = 6,842, *p* = 0.015, η^2^ = 0.028, 95% CI [0,000, 0.279].

### Pre-injury Variables

**FIGURE 4:**
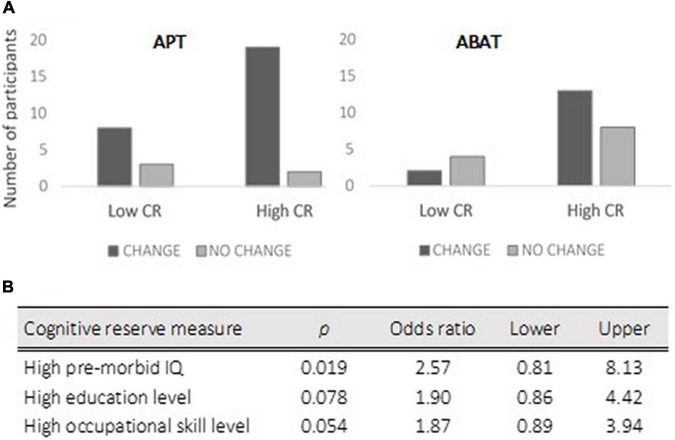
The upper chart of the figure **(A)** presents the distribution of patients in the CHANGE vs. NO CHANGE groups when patients were divided into high vs. low cognitive reserve (CR) for each treatment (APT, Attention Process Training; ABAT, Activity-Based Attention Training). The lower part **(B)** presents the logistic regression analysis results for cognitive reserve measures with a 95% confidence interval.

More APT patients with higher cognitive reserve displayed CHANGE [Pearson *X*^2^ (1, *N* = 42) = 4,725, *p* = 0.030; ES V = 0.34; OR = 0.34 for NO CHANGE/CHANGE with 95% CI (0.832, 10.596) and *p* = 0.030]. Crosstabulation revealed a significant linear-by-linear association of 5,213 (*p* = 0.022) between high premorbid IQ (Matrices from WAIS-III) and CHANGE after APT, but not for education or occupational skill level (Figure 4).

There were no significant differences between groups regarding age, gender, family status, length of education, or occupational skills level (Table 1).

## Discussion

The present results indicate the advantage of the APT training for stroke patients early after brain injury, particularly for right hemisphere unifocal lesions (moderate ES). For TBI patients, the type of attention training seemed to have little or no influence on the outcome. The apparent advantage of APT treatment suggests the assumption that systematic attention training might target brain mechanisms for supervising and coordinating higher-order attention functions (Mengotti et al., 2020). Imaging studies are needed to analyze the putative mechanisms within the attentional networks. The present results strongly indicate that patients with right-hemispheric stroke need to receive systematic cognitive training of attention, although the guidelines do not emphasize specifically the consequence of etiology (Haskins et al., 2013; Cicerone et al., 2019; Loetscher et al., 2019; Spaccavento et al., 2019).

The influence of cognitive variables was limited. Patients with higher cognitive reserve (moderate ES) benefitted more from APT treatment. Among the variables measuring initial cognitive functioning, only the most complex subtest measuring verbal cognitive flexibility (CWIT 4) contributed to a positive outcome, i.e., CHANGE (moderate ES). Better executive functions, such as inhibition, switching, and mental flexibility reflected by CWIT 4, might promote treatment effect. Earlier studies have identified executive functions and cognitive reserve as predictors of improved intervention outcomes (Hanks et al., 2008; Umarova et al., 2019).

The inconclusive results regarding APT after TBI are discouraging. Still, they are in line with the metanalysis of attention training after ABI (Rohling et al., 2009) with mean ES 0.06 for TBI studies and ES 0.48 for stroke. The importance of injury localization (for motor and language functions) and functional connectivity, particularly in white matter (for memory and attention), has been demonstrated in stroke (Corbetta et al., 2015). Due to the biomechanical forces affecting the brain during TBI, patients exhibit multifocal injuries and diffuse axonal injury by shearing, leading to multiple disturbances in functional connectivity (Johnson et al., 2013). Consequently, connectivity disruptions might hamper mechanisms promoting training effects for APT. The specific recommendations of the INCOG group for TBI support training in metacognitive strategies applied directly to everyday attentional difficulties (Ponsford et al., 2014).

A third of the participants belonged to NO CHANGE despite expected spontaneous recovery and rehabilitation effects in this early stage after ABI (Hochstenbach et al., 2003; Barker-Collo et al., 2010). None of our variables was related to this finding. Other studies have found similar proportions of poor recovery for neglect and aphasia post-stroke (Marchi et al., 2017) and motor functions (Prabhakaran et al., 2008; Winters et al., 2015). Identifying factors behind poor recovery is critical and underlie the choice of adequate treatment.

Thus, the special effect of APT training on stroke patients with unilateral right-hemispheric lesions and the moderate effect of mental flexibility and cognitive reserve might suggest two different pathways leading to improvement for attention training: one directly affecting attentional functioning in crucial brain structures and another, more compensatory, utilizing preserved higher cognitive functions.

These results should be interpreted with caution. As in our study, small sample sizes increase the possibility for type II error. Subject heterogeneity is an issue in brain injury research, and we followed the recommendations for selection criteria for treatment studies (Cicerone, 2012).

Due to our strict selection criteria, our group was relatively homogeneous regarding cognitive functioning, i.e., mild to moderate attention impairment with no other significant cognitive disturbances. Also, the lack of influence of some demographic factors, as found in earlier studies (Leary et al., 2018) may reflect the relative homogeneity of our participant group. But to obtain the present group, we needed to screen 626 potential participants (Markovic et al., 2017a).

The rationale for using ANOVA was based on our clinical interests to improve the accuracy of treatment planning for optimal treatment outcomes. Therefore, we should view the results from the present study as tentative. Furthermore, as the study was exploratory, future studies with larger samples are needed to validate the findings.

The SPC method is relatively new in rehabilitation. The procedure in our RCT study was modified (Markovic, 2017) by reducing the number of measurement points and developing an alternative approach to estimate baseline. These modifications provided both individual and group charts allowing to monitor the treatment process in real-time. Thus, the use of SPC allowed us to combine the qualitative advantages of single-case experimental design (SCED) (Krasny-Pacini and Evans, 2018) with the rigor of an RCT design, offering a deeper understanding of the process of rehabilitation. In addition, the combination of the larger number of participants than in SCED and more observations than in RCT allowed us to discern improvers (CHANGE) from non-improvers (NO-CHANGE) on an individual level and to identify predisposing factors for treatment outcome. Another advantage of SPC is that it may be used for individual patients as part of an individual rehabilitation plan for monitoring and continuous evaluation akin to SCED. Still, it offers statistical rules for interpreting the charts (Tennant et al., 2007). The debate of SPC use revolves around several areas (Woodall, 2000).

One of the critical key issues is the idea of control charts as repeated hypothesis testing. Whereas some scholars argue that it resembles a continuous statistical hypothesis testing system, others criticize this view and put forth that natural processes are dynamic and do not represent stable systems. Consequently, in practice, the use of control charts should not be based on a well-defined finite population. Another issue is the role of various rules for the detection of alarms/signals. Commonly, 3 SD from the mean value is used as a default rule. Any point outside this limit is considered an alarm. Also, as applied in the present paper, other rules may be used. An advantage of applying different rules than 3 SD from the mean value is that more types of significant behavior can be detected. However, statistically, this also has the consequence of increasing type II error, thus the risk of falsely assuming that a real change in patient performance has happened.

The choice of the control treatment is central (Sigmundsdottir et al., 2016). We have selected ABAT as an active control group, despite being based on a different model. APT is a direct structured neuropsychological intervention on the level of body function (Stucki et al., 2019) improving performance on training tasks and immediate measures of global attention (Cicerone et al., 2019). The ABAT training, in contrast, is an occupational therapy intervention comparable to the Cognitive Orientation to Occupational Performance (CO-OP) (Polatajko et al., 2012; McEwen et al., 2015) aiming at functional skills training on activity level, as defined by the International Classification of Functioning and Health (ICF) domains of body functions with activity and participation (Sigmundsdottir et al., 2016), and the incorporation of metacognitive strategies to improve performance on trained tasks.

These theoretical differences are also apparent in our measurement methods.

We lack instruments for the level of activity and participation to measure suitably the effects of the ABAT. The relationship between neuropsychological test results and activity in real-world functioning has been previously discussed (Chaytor and Schmitter-Edgecombe, 2003). Methodological issues are probably also reflected in the lack of relationship between treatment effects and other neuropsychological tests of attention, working memory and episodic memory. However, the lack of results might reflect that these tests are different aspects of attention than those trained by the APT method.

Therefore, given the homogeneity of our study sample, particularly the limited sample size of TBI patients and methodological issues, the results should be interpreted with caution. Using the SPC method, multicentre studies within rehabilitation medicine are needed to obtain larger groups, allowing for detailed subgroup analysis.

## Conclusion and Clinical Implications

The application of the SPC method allowed us to delineate a more detailed process of attention training. Our explorative results strengthen the importance of considering the physiological nature of the brain insult and premorbid cognitive functioning when considering the appropriate choice of intervention for restitution of attention after ABI. The results of the present study have several clinical implications. It is crucial to provide systematic training of attention to patients with right-hemispheric stroke in an early phase of recovery. On the other hand, training for TBI patients should focus on compensatory cognitive strategies and metacognitive training. Understanding prognostic factors are crucial to maximizing resource allocation and the effectiveness of rehabilitative interventions to enhance outcomes following stroke and TBI.

## Data Availability Statement

The original contributions presented in the study are included in the article/Supplementary Material, further inquiries can be directed to the corresponding author.

## Ethics Statement

This work is part of a clinical RCT trial (registration no. NCT02091453) (Bartfai et al., 2014) comparing two attention interventions. Data collection was carried out in concordance with the Helsinki Declaration and approved by the Karolinska Institutet Ethical Committee (registration no 2007/1363-31). The patients/participants provided their written informed consent to participate in this study.

## Author Contributions

AB and GM were responsible for study design. GM and ME were responsible for statistical analyses and prepared the figures. All authors were responsible for the preparation of the manuscript.

## Conflict of Interest

The authors declare that the research was conducted in the absence of any commercial or financial relationships that could be construed as a potential conflict of interest.

## Publisher’s Note

All claims expressed in this article are solely those of the authors and do not necessarily represent those of their affiliated organizations, or those of the publisher, the editors and the reviewers. Any product that may be evaluated in this article, or claim that may be made by its manufacturer, is not guaranteed or endorsed by the publisher.

## Supplementary Material

Supplementary Figure 1 I-diagrams (*n* = 59) for PASAT during attention training (20 h) at eight measurement points, including a centre line and upper and lower control limits, respectively, per plot of individual observations. PASAT raw scores range from 10 to 60 points. Treatment outcome is defined as CHANGE/NO CHANGE according to criteria of statistical process control. I-diagrams are organized to fulfil statistical criteria, represented by a square for each measurement point where the criteria are met.

## References

[B1] Arntzen C. Borg T. Hamran T. (2015). Long-term recovery trajectory after stroke: an ongoing negotiation between body, participation and self. *Disabil. Rehabil.* 37 1626–1634. 10.3109/09638288.2014.972590 25318537

[B2] Barker-Collo S. Feigin V. Lawes C. Senior H. Parag V. (2010). Natural history of attention deficits and their influence on functional recovery from acute stages to 6 months after stroke. *Neuroepidemiology* 35 255–262. 10.1159/000319894 20881428

[B3] Barman A. Chatterjee A. Bhide R. (2016). Cognitive impairment and rehabilitation strategies after traumatic brain injury. *Indian J. Psychol. Med.* 38 172–181. 10.4103/0253-7176.183086 27335510 PMC4904751

[B4] Bartfai A. Markovic G. Sargenius Landahl K. Schult M. L. (2014). The protocol and design of a randomised controlled study on training of attention within the first year after acquired brain injury. *BMC Neurol.* 14:102. 10.1186/1471-2377-14-102 24885585 PMC4018266

[B5] Barulli D. Stern Y. (2013). Efficiency, capacity, compensation, maintenance, plasticity: emerging concepts in cognitive reserve. *Trends Cogn. Sci.* 17 502–509. 10.1016/j.tics.2013.08.012 24018144 PMC3840716

[B6] Benneyan J. C. Lloyd R. C. Plsek P. E. (2003). Statistical process control as a tool for research and healthcare improvement. *Qual. Saf. Health Care* 12 458–464. 10.1136/qhc.12.6.458 14645763 PMC1758030

[B7] Callahan C. D. Barisa M. T. (2005). Statistical process control and rehabilitation outcome: the single-subject design reconsidered. *Rehabil. Psychol.* 50 24–33. 10.1037/0090-5550.50.1.24

[B8] Chaytor N. Schmitter-Edgecombe M. (2003). The ecological validity of neuropsychological tests: a review of the literature on everyday cognitive skills. *Neuropsychol. Rev.* 13 181–197. 10.1023/B:NERV.0000009483.91468.fb 15000225

[B9] Cicerone K. D. (2012). Facts, theories, values: shaping the course of neurorehabilitation. the 60th John Stanley Coulter memorial lecture. *Arch. Phys. Med. Rehabil.* 93 188–191. 10.1016/j.apmr.2011.12.003 22289226

[B10] Cicerone K. D. Dahlberg C. Malec J. F. Langenbahn D. M. Felicetti T. Kneipp S. (2005). Evidence-based cognitive rehabilitation: updated review of the literature from 1998 through 2002. *Arch. Phys. Med. Rehabil.* 86 1681–1692. 10.1016/j.apmr.2005.03.024 16084827

[B11] Cicerone K. D. Goldin Y. Ganci K. Rosenbaum A. Wethe J. V. Langenbahn D. M. (2019). Evidence-based cognitive rehabilitation: systematic review of the literature from 2009 through 2014. *Arch Phys. Med. Rehabil.* 100 1515–1533. 10.1016/j.apmr.2019.02.011 30926291

[B12] Cohen J. (1988). *Statistical Power Analysis for the Behavioral Sciences*, Vol. 2. Hillsdale, NJ: L. Erlbaum Associates.

[B13] Corbetta M. Ramsey L. Callejas A. Baldassarre A. Hacker C. D. Siegel J. S. (2015). Common behavioral clusters and subcortical anatomy in stroke. *Neuron* 85 927–941. 10.1016/j.neuron.2015.02.027 25741721 PMC4646844

[B14] Delis D. C. Kaplan E. Kramer A. F. (2001). *Delis-Kaplan Executive Function System: Examiner’s Manual.* Agra: The Psychological Corporation.

[B15] Dymowski A. R. Ponsford J. L. Willmott C. (2016). Cognitive training approaches to remediate attention and executive dysfunction after traumatic brain injury: a single-case series. *Neuropsychol. Rehabil.* 26 866–894. 10.1080/09602011.2015.1102746 26493353

[B16] Goverover Y. Genova H. Smith A. Chiaravalloti N. Lengenfelder J. (2017). Changes in activity participation following traumatic brain injury. *Neuropsychol. Rehabil.* 27 472–485. 10.1080/09602011.2016.1168746 27043964

[B17] Gronwall D. M. (1977). Paced auditory serial-addition task: a measure of recovery from concussion. *Percept. Mot. Skills* 44 367–373. 10.2466/pms.1977.44.2.367 866038

[B18] Hanks R. A. Millis S. R. Ricker J. H. Giacino J. T. Nakese-Richardson R. Frol A. B. (2008). The predictive validity of a brief inpatient neuropsychologic battery for persons with traumatic brain injury. *Arch. Phys. Med. Rehabil.* 89 950–957. 10.1016/j.apmr.2008.01.011 18452745

[B19] Haskins M. E. Cicerone K. D. Dams-O’Connor K. Langenbahn D. M. Shapiro-Rosenbaum A. (2013). in *Cognitive Rehabilitation Manual. Translating Evidence-based Recommendations into Practice*, ed. Trexler L. (Atlanta, GA: ACRM Publishing). (BI-ISIG).

[B20] Hochstenbach J. B. den Otter R. Mulder T. W. (2003). Cognitive recovery after stroke: a 2-year follow-up. *Arch. Phys. Med. Rehabil.* 84 1499–1504. 10.1016/s0003-9993(03)00370-8 14586918

[B21] Johnson V. E. Stewart W. Smith D. H. (2013). Axonal pathology in traumatic brain injury. *Exp. Neurol.* 246 35–43. 10.1016/j.expneurol.2012.01.013 22285252 PMC3979341

[B22] Krasny-Pacini A. Evans J. (2018). Single-case experimental designs to assess intervention effectiveness in rehabilitation: a practical guide. *Ann. Phys. Rehabil. Med.* 61 164–179. 10.1016/j.rehab.2017.12.002 29253607

[B23] Lakens D. (2013). Calculating and reporting effect sizes to facilitate cumulative science: a practical primer for t-tests and ANOVAs. *Front. Psychol.* 4:863. 10.3389/fpsyg.2013.00863 24324449 PMC3840331

[B24] Leary J. B. Kim G. Y. Bradley C. L. Hussain U. Z. Sacco M. Bernad M. (2018). The association of cognitive reserve in chronic-phase functional and neuropsychological outcomes following traumatic brain injury. *J. Head Trauma Rehabil.* 33 E28–E35. 10.1097/htr.0000000000000329 28731870 PMC5752441

[B25] Lezak M. D. Howieson D. B. Bigler E. D. Tranel D. (2012). *Neuropsychological Assessment*, 5th Edn. Oxford: Oxford University Press.

[B26] Loetscher T. Potter K. J. Wong D. das Nair R. (2019). Cognitive rehabilitation for attention deficits following stroke. *Cochrane Database Syst. Rev.* 2013:CD002842. 10.1002/14651858.CD002842.pub3 31706263 PMC6953353

[B27] Mahoney F. I. Barthel D. W. (1965). Functional evaluation: the barthel index. *Md. State Med. J.* 14 61–65. 14258950

[B28] Marchi N. A. Ptak R. Di Pietro M. Schnider A. Guggisberg A. G. (2017). Principles of proportional recovery after stroke generalize to neglect and aphasia. *Eur. J. Neurol.* 24 1084–1087. 10.1111/ene.13296 28585297

[B29] Markovic G. (2017). *Acquired Brain Injury and Evaluation of Intensive Training of Attention in Early Neurorehabilitation : Statistical Evaluation and Qualitative Perspectives.* Stockholm: Karolinska Institutet.

[B30] Markovic G. Schult M. L. Bartfai A. (2017a). The effect of sampling bias on generalizability in intervention trials after brain injury. *Brain Inj.* 31 9–15. 10.1080/02699052.2016.1206213 27819515

[B31] Markovic G. Schult M. L. Bartfai A. Elg M. (2017b). Statistical process control: a feasibility study of the application of time-series measurement in early neurorehabilitation after acquired brain injury. *J. Rehabil. Med.* 49 128–135. 10.2340/16501977-2172 27904913

[B32] Markovic G. Schult M. L. Elg M. Bartfai A. (2019). Beneficial effects of early attention process training after acquired brain injury: a randomized controlled trial. *J. Rehabil. Med.* 52:jrm00011. 10.2340/16501977-2628 31742648

[B33] Markovic G. Schult M. L. Elg M. Bartfai A. (2020). Beneficial effects of early attention process training after acquired brain injury: a randomized controlled trial. *J. Rehabil. Med.* 52:jrm00011. 10.2340/16501977-2628 31742648

[B34] McEwen S. Polatajko H. Baum C. Rios J. Cirone D. Doherty M. (2015). Combined cognitive-strategy and task-specific training improve transfer to untrained activities in subacute stroke: an exploratory randomized controlled trial. *Neurorehabil. Neural Repair* 29 526–536. 10.1177/1545968314558602 25416738 PMC4440855

[B35] Mengotti P. Käsbauer A. S. Fink G. R. Vossel S. (2020). Lateralization, functional specialization, and dysfunction of attentional networks. *Cortex* 132 206–222. 10.1016/j.cortex.2020.08.022 32998061

[B36] Montgomery D. C. (2012). *Statistical Quality Control*, 7th Edition International Student Edn. Hoboken, NJ: Wiley Global Education.

[B37] Nasr N. Mawson S. Wright P. Parker J. Mountain G. (2016). Exploring the experiences of living with stroke through narrative: stroke survivors’. Perspectives. *Glob. Qual. Nurs. Res.* 3:2333393616646518. 10.1177/2333393616646518 28462337 PMC5342850

[B38] Oldenburg C. Lundin A. Edman G. Nygren-de Boussard C. Bartfai A. (2016). Cognitive reserve and persistent post-concussion symptoms–a prospective mild traumatic brain injury (mTBI) cohort study. *Brain Inj.* 30 146–155. 10.3109/02699052.2015.1089598 26618716

[B39] Polatajko H. J. McEwen S. E. Ryan J. D. Baum C. M. (2012). Pilot randomized controlled trial investigating cognitive strategy use to improve goal performance after stroke. *Am. J. Occup. Ther.* 66 104–109. 10.5014/ajot.2012.001784 22389945

[B40] Ponsford J. Bayley M. Wiseman-Hakes C. Togher L. Velikonja D. McIntyre A. (2014). INCOG recommendations for management of cognition following traumatic brain injury, part II: attention and information processing speed. *J. Head Trauma Rehabil.* 29 321–337. 10.1097/htr.0000000000000072 24984095

[B41] Prabhakaran S. Zarahn E. Riley C. Speizer A. Chong J. Y. Lazar R. M. (2008). Inter-individual variability in the capacity for motor recovery after ischemic stroke. *Neurorehabil. Neural Repair* 22 64–71. 10.1177/1545968307305302 17687024

[B42] Raskin S. A. (2011). “Introduction: current approaches to rehabilitation,” in *Neuroplasticity and Rehabilitation*, ed. Raskin S. A. (New York, NY: The Guilford Press), 1–9.

[B43] Rohling M. L. Faust M. E. Beverly B. Demakis G. (2009). Effectiveness of cognitive rehabilitation following acquired brain injury: a meta-analytic re-examination of Cicerone’s (2000, 2005) systematic reviews. *Neuropsychology* 23 20–39. 10.1037/a0013659 19210030

[B44] Rosti-Otajärvi E. Mäntynen A. Koivisto K. Huhtala H. Hämäläinen P. (2013). Patient-related factors may affect the outcome of neuropsychological rehabilitation in multiple sclerosis. *J. Neurol. Sci.* 334 106–111. 10.1016/j.jns.2013.07.2520 23968849

[B45] Ruff D. M. Niemann H. Allen C. C. Farrow C. E. Wylie T. (1992). The ruff 2 and 7 selective attention test. *Percept. Mot. Skills* 75, 1311–1319. 10.2466/pms.1992.75.3f.1311 1484803

[B46] Sargénius Landahl K. (2021). *Evaluation of Attention Training After Acquired Brain Injury – An Occupational Perspective. (Lic D Rehabilitation Medicine).* Ann Arbor, MI: ProQuest Dissertations Publishing.

[B47] Sigmundsdottir L. Longley W. A. Tate R. L. (2016). Computerised cognitive training in acquired brain injury: a systematic review of outcomes using the International Classification of Functioning (ICF). *Neuropsychol. Rehabil.* 26 673–741. 10.1080/09602011.2016.1140657 26965034

[B48] Skoglund E. Westerlind E. Persson H. C. Sunnerhagen K. S. (2019). Self-perceived impact of stroke: a longitudinal comparison between one and five years post-stroke. *J. Rehabil. Med.* 51 660–664. 10.2340/16501977-2595 31478056

[B49] Sohlberg M. M. Mateer C. A. (1987). Effectiveness of an attention-training program. *J. Clin. Exp. Neuropsychol.* 9 117–130. 10.1080/01688638708405352 3558744

[B50] Sohlberg M. M. Mateer C. A. (1989). *Introduction to Cognitive Rehabilitation.* New York, NY: The Guilford Press.

[B51] Sohlberg M. M. Turkstra L. S. (2011). *Optimizing Cognitive Rehabilitation. Effective Instructional Methods.* New York, N Y: The Guilford Press.

[B52] Spaccavento S. Marinelli C. V. Nardulli R. Macchitella L. Bivona U. Piccardi L. (2019). Attention deficits in stroke patients: the role of lesion characteristics, time from stroke, and concomitant neuropsychological deficits. *Behav. Neurol.* 2019:7835710. 10.1155/2019/7835710 31263512 PMC6556322

[B53] Stephens J. A. Williamson K. N. Berryhill M. E. (2015). Cognitive rehabilitation after traumatic brain injury: a reference for occupational therapists. *OTJR (Thorofare N J)* 35 5–22. 10.1177/1539449214561765 26623474 PMC6730543

[B54] Stucki G. Pollock A. Engkasan J. P. Selb M. (2019). How to use the international classification of functioning, disability and health as a reference system for comparative evaluation and standardized reporting of rehabilitation interventions. *Eur. J. Phys. Rehabil. Med.* 55 384–394. 10.23736/s1973-9087.19.05808-8 30990004

[B55] Tennant R. Mohammed M. A. Coleman J. J. Martin U. (2007). Monitoring patients using control charts: a systematic review. *Int. J. Qual. Health Care* 19 187–194. 10.1093/intqhc/mzm015 17545672

[B56] Thor J. Lundberg J. Ask J. Olsson J. Carli C. Harenstam K. P. (2007). Application of statistical process control in healthcare improvement: systematic review. *Qual. Saf. Health Care* 16 387–399. 10.1136/qshc.2006.022194 17913782 PMC2464970

[B57] Tornås S. Stubberud J. Solbakk A. K. Evans J. Schanke A. K. Løvstad M. (2019). Moderators, mediators and nonspecific predictors of outcome after cognitive rehabilitation of executive functions in a randomised controlled trial. *Neuropsychol. Rehabil.* 29 844–865. 10.1080/09602011.2017.1338587 28651477

[B58] Umarova R. M. Sperber C. Kaller C. P. Schmidt C. S. M. Urbach H. Klöppel S. (2019). Cognitive reserve impacts on disability and cognitive deficits in acute stroke. *J. Neurol.* 266 2495–2504. 10.1007/s00415-019-09442-6 31254064

[B59] Wallenbert I. Jonsson H. (2005). Waiting to get better: a dilemma regarding habits in daily occupations after stroke. *Am. J. Occup. Ther.* 59 218–224. 10.5014/ajot.59.2.218 15830622

[B60] Wilson B. A. Cockburn J. Baddeley A. (2000). *The Rivermead Behavioral Memory Test.* Bury St Edmunds: Thames Valley Test Company.

[B61] Winters C. van Wegen E. E. Daffertshofer A. Kwakkel G. (2015). Generalizability of the proportional recovery model for the upper extremity after an ischemic stroke. *Neurorehabil. Neural Repair* 29 614–622. 10.1177/1545968314562115 25505223

[B62] Woodall W. H. (2000). Controversies and contradictions in statistical process control. *J. Qual. Technol.* 32 341–350.

[B63] Zigmond A. S. Snaith R. P. (1983). The hospital anxiety and depression scale. *Acta Psychiatr. Scand.* 67 361–370. 10.1111/j.1600-0447.1983.tb09716.x 6880820

