# Supplementary material for: Predicting Outcome for Early Attention Training After Acquired Brain Injury
Source: Front Hum Neurosci. 2022 May 18;16:767276. doi: 10.3389/fnhum.2022.767276 (PMC9159897; doi:10.3389/fnhum.2022.767276)
Supplement: Supplementary file 1 [file Data_Sheet_1.pdf]

Appendix 1

Cognitive reserve measures

Measures for cognitive reserve were based on educational level, occupational skill level and estimated premorbid IQ.

Educational level was classified into four categories according to the International Standard Classification of Educational Degrees, ISCED-2011 ((UNESCO), 2012). The categories are (1) elementary school comprising Primary and Lower secondary Education since the first nine years of education are mandatory in Sweden, (2) Upper secondary Education (12yrs), (3) Bachelor's or equivalent level (13-15yrs), (4) Master's or equivalent level and above (≥16yrs). Participants were categorized according to highest attained educational level.

Occupational skill level was classified into four broad skill levels in order of complexity and according to the International Standard Classification of Occupation, ISCO-08 (International Standard Classification of Occupation, 2012). The latest occupation was used for classification. The four levels are *(Level 1)* occupations involving simple or routine tasks, such as bartender and cashier, *(Level 2)* occupations involving operating equipment or machinery, such as secretary and carpenter, *(Level 3)* occupations involving complexity in task performance and requiring specialization, such as nurse and programmer, and *(Level 4)* occupations involving complex decision-making and problem-solving with extensive body of knowledge, such as medical practitioners and managers.

Premorbid IQ was estimated from the subtest Matrices from WAIS-III. The test measures reasoning skills and abstract thinking and is considered a 'hold'-test rarely affected by injury (Lezak et al., 2012).

To test the association between CR and CHANGE, the variables were dichotomized (Table 1) and analysed with one-way ANOVA for identification of potential significant differences among the means in the three variables. The study dichotomized the three variables and performed logistic regression analysis for each variable separately in order test the hypothesis that cognitive reserve is associated to CHANGE. Cut-off values for high educational level was ≥13 years, low occupational skill level was defined as level 3-4 according to ISCO-08, and cut-off for high premorbid IQ was ≥13 scaled scores.

Table 1. Dichotomized estimates for cognitive reserve according to the type of treatment and treatment outcome (CHANGE vs NO CHANGE). Results are presented in number (n) and distribution (%) of participants.

Variable	Total sample	APT
		CHANGE
	(n=59)	*(n=27)*
Education [a]		
≤12 years	17 (28)	8 (30)
≥13 years	43 (72)	19 (70)
Matrices (WAIS-III) [b], n (%)		
7-12 Scaled Score	38 (63)	15 (56)
13+ Scaled Score	21 (35)	12 (44)
Occupational skills level [c], n (%)		
Simple (Level 1-2)	15 (25)	7 (26)
Complex (Level 3-4)	45 (75)	20 (74)
Cognitive reserve (CR) [d], n (%)		
Low CR	18 (30)	8 (30)
High CR	42 (70)	19 (70)

Abbreviations: APT, Attention Process Training; ABAT, Activity-based attention training; n, number.
[a] Highest attained level of education following ISCED-2011 presented as years of education, ≤12 yrs. comprising Level 1 and 2, ≥13 years; [b] WAIS-III, The Wechsler Intelligence Scale- III, expressed in Scaled Score where results ≥13 represent results above normal distribution in healthy subjects; [c] Simple occupational skill level corresponds to Level 1-2 as defined by the ISCO-08, and Complex occupational skill level corresponds to Level 3-4.
