# Supplementary figures and images for: Predicting Outcome for Early Attention Training After Acquired Brain Injury
Source: Front Hum Neurosci. 2022 May 18;16:767276. doi: 10.3389/fnhum.2022.767276 (PMC9159897; doi:10.3389/fnhum.2022.767276)

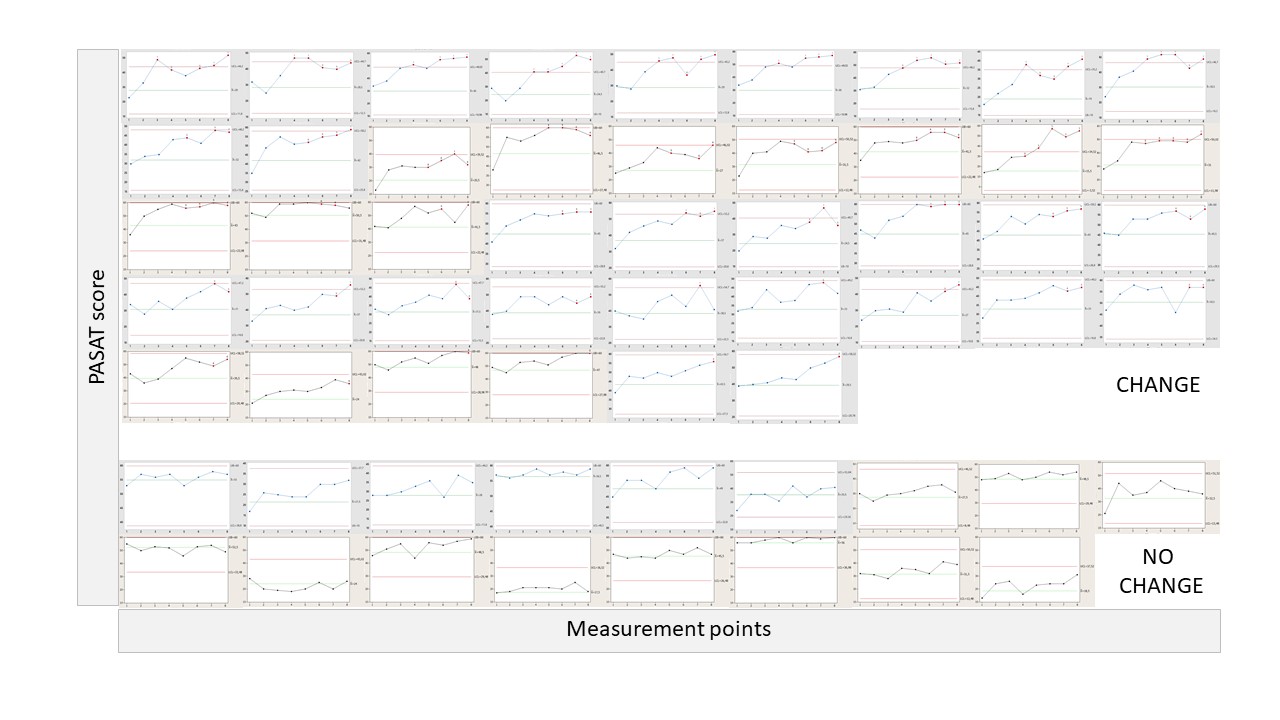

Supplement: Supplementary Figure 1 — I-diagrams (n = 59) for PASAT during attention training (20 h) at eight measurement points, including a centre line and upper and lower control limits, respectively, per plot of individual observations. PASAT raw scores range from 10 to 60 points. Treatment outcome is defined as CHANGE/NO CHANGE according to criteria of statistical process control. I-diagrams are organized to fulfil statistical criteria, represented by a square for each measurement point where the criteria are met. [file Image_1.jpeg]
